# Dose-Dependent Application of Straw-Derived Fulvic Acid on Yield and Quality of Tomato Plants Grown in a Greenhouse

**DOI:** 10.3389/fpls.2021.736613

**Published:** 2021-10-11

**Authors:** Peijia Zhang, Hongjia Zhang, Guoqing Wu, Xiaoyuan Chen, Nazim Gruda, Xun Li, Jinlong Dong, Zengqiang Duan

**Affiliations:** ^1^State Key Laboratory of Soil and Sustainable Agriculture, Institute of Soil Science, Chinese Academy of Sciences, Nanjing, China; ^2^University of Chinese Academy of Sciences, Beijing, China; ^3^Nutrition and Health Research Institute, COFCO, Beijing, China; ^4^Institute of Crop Science and Resource Conservation, Division of Horticultural Sciences, University of Bonn, Bonn, Germany

**Keywords:** humic substances, nutritional quality, plant biomass, seed germination, soil organic matter

## Abstract

Fulvic acids are organic compounds widely distributed in soils, and the application of fulvic acids is thought to increase crop yield and quality. However, the effects vary among various sources and doses of fulvic acids and environmental and growth conditions of crops. Here, we investigated the effects of bioresource-derived (corn straw) fulvic acids on plant production and quality of tomato plants and soil chemical properties in soil cultures while experiments on seed germination and hydroponics were conducted to explore the underlying mechanism. Base dressing with 2.7 g kg^–1^ increased the yield of tomato by 35.0% at most as increased fruit number. Fulvic acids increased the concentrations of minerals, such as Ca, Fe, and Zn and the concentrations of citric, malic, and some amino acids in berries of tomato but did not affect the concentrations of soluble sugars and aromatic substances in tomato fruits. Similarly, fulvic acids at 80–160 mg L^–1^ increased germination rate, growth vigor, and radicle elongation of tomato seeds while it increased plant biomass, concentrations of nutrients, and root length of tomato plants in hydroponics to the greatest extent in general. The increases in yield and quality can be attributed to the improvement in root growth and, thus, increased nutrient uptake. In addition, the base application of fulvic acids improved soil cation exchange capacity and soil organic matter to an extent. In conclusion, base dressing and the addition into solution of fulvic acids at moderate doses facilitate root growth and nutrient uptake and, thus, vegetable production and quality; therefore, fulvic acids can be an effective component for designing new biofertilizers for sustainable agricultural production.

## Introduction

Fulvic acids are one portion of soil humic substances based on the solubility in strong acid and base solutions; the other two main portions are humic acids and humins (Hayes, 2006; Ahmad et al., 2018). Fulvic acids consist of a group of soluble organic compounds widely distributed in nature and are one of the critical components of soil organic substances (Piccolo, 2002; Qin et al., 2016; Shah et al., 2018). These organic compounds contain many active functional groups, such as carbonyl, carboxyl, hydroxyl, phenolic hydroxyl, and quinone, and these are capable of chelating and exchanging anions or ions (Calvo et al., 2014; de Melo et al., 2016; Zhang et al., 2020). The mechanisms by which humic-like substances improve plant growth can be attributed to the increased ability of regulating membrane permeability and intracellular signaling, thus increasing root growth (Blomster et al., 2011), increasing concentrations of chlorophyll and photosynthetic activity (Haghighi et al., 2012), and activating carbon and nitrogen metabolism (Jannin et al., 2012). In addition, the biochemical fulvic acids encompass amino acids, vitamins, trace elements, and hormones, and all those compounds can stimulate cell division, root growth, and nutrient uptake and improve the antistress ability of plants and, thus, promote the growth and, thus, yield of crops (De Pascale et al., 2018; Shah et al., 2018; Qin and Leskovar, 2020). For example, fulvic acids are demonstrated to relieve Pb toxicity to plants by reducing its uptake, thus alleviating various morphological, physiological, and biochemical functions of plants (Shahid et al., 2012).

In recent decades, numerous studies confirm the effectiveness of fulvic acids in agriculture. Fulvic acids are demonstrated to increase the yield and quality of cotton plants, soil fertility, fertilizer-use efficiency, and net profit (Geng et al., 2020). The application of fulvic acids alleviates the damage on wheat plants under salinity stress by improving antioxidant-defense systems, thus increasing growth and production (Elrys et al., 2020). With respect to vegetable production, fulvic acids increase the size and number of tomatoes while decreasing the incidence of cracking and blossom-end rotting (Suh et al., 2014). As a result of increased plant growth, fulvic acids are recommended as an essential constituent to achieve the goal of high yield and quality and sustainable agriculture in horticulture particularly (Olk et al., 2018).

However, due to the complex composition of fulvic acids, in-depth trials have not been not comprehensively investigated, especially the effects of bioresource fulvic acids on the yield and quality of vegetables and the underlying mechanisms. Bioresource fulvic acids are renewable and recyclable, and thus, the popular agricultural fulvic acids in recent decades are considerable for sustainable agriculture (Quilty and Cattle, 2011; Rose et al., 2014).

This study investigates the effects of straw-derived fulvic acids on vegetable plants. We carried out soil experiments, seed germination, and hydroponic cultures to explore the effects of various doses and application patterns on the production, yield, and quality of tomato plants and the underlying mechanisms that potentially facilitate the design of new biofertilizers by using fulvic acids.

## Materials and Methods

Bioresource-derived fulvic acids, abbreviated as Zhongliang or ZL in the study, were produced by the national company COFCO Nutrition and Health Research Institute, Beijing, China (Table 1). ZL fulvic acids are derived from corn straw. The lignocellulosic corn straws were pretreated by continuous steam explosion and then hydrolyzed by a series of enzymes (cellulase, hemicellulase, and β-glucosidase) to get a sugar solution. The sugar solution was fermented by using a pentose/hexose co-fermentation strain (C5 strains, Green Tech America, United States) to get the mature fermented mash. Then, the mash was rectified to obtain biofuel ethanol from the top of the rectification tower while the residual mash was obtained from the bottom of the rectification tower. The residual mash was separated from the supernatant and then evaporated, concentrated, and tube-bundle-dried to get the final fulvic acids.

**TABLE 1:** The chemical properties of fulvic acids of Zhongliang used in this study.

**Items**	**Value**	**Items**	**Value**	**Items**	**Value**
Fulvic acids	69.5%	Ca (g kg^–1^)	9.25	As (μg g^–1^)	2.36
pH	3.94	Mg (g kg^–1^)	5.22	Cd (μg g^–1^)	0.23
Water content	6.11%	Fe (μg g^–1^)	1123	Cr (μg g^–1^)	18.9
Ach content	19.1%	Mn (μg g^–1^)	135	Hg (μg g^–1^)	0
Total C	30.3%	Cu (μg g^–1^)	2.54	Pb (μg g^–1^)	2.00
Total N	5.63%	Zn (μg g^–1^)	52.3		
Total P (P_2_O_5_)	0.26%				
Total K (K_2_O)	3.09%				

### Experiment 1: Soil Culture of Tomato Plants

Experiment 1 was a soil culture to explore the effects of fulvic acids on the yield and quality of a fruit vegetable and soil fertility (Supplementary Figure 1). A randomized complete block design with 11 treatments consisted of various methods of fulvic acid application, i.e., top dressing, foliar, and base dressing (Table 2). Another commercial fulvic acid produced by QuanlinJiayou Co., Ltd. (Jiayou or JY) that was the most effective and popular product in China was used to compare with our product and to demonstrate the effectiveness of our Zhongling (ZL) fulvic acids. Five replicates were set for each treatment.

**TABLE 2:** The treatments of soil culture for tomato growth (Experiment 1).

**Fertilization**	**Chemicals**	**Rates**	**Abbreviation**
Control	Water	Local practice	Control
Top dressing	ZL fulvic	0.3 g kg^–1^	ZL Top
Foliar application	acids	100 mg L^–1^	ZL Foliar
Top dressing + Foliar		0.3 g kg^–1^ + 100 mg L^–1^	ZL T + F
Top dressing	JY fulvic	0.3 g kg^–1^	JY Top
Foliar application	acids	100 mg L^–1^	JY Foliar
Top dressing + Foliar		0.3 g kg^–1^ + 100 mg L^–1^	JY T + F
Base dressing	ZL fulvic	0.3 g kg^–1^	Base 0.3
Base dressing	acids	0.9 g kg^–1^	Base 0.9
Base dressing		2.7 g kg^–1^	Base 2.7
Base dressing		8.1 g kg^–1^	Base 8.1

The soil was collected from the top 0–20 cm in a high tunnel vegetable farm managed for 18 years in Shanghai, China (Table 3). In this area of the Yangtze River Delta, rice was produced for several hundred years before, and then the fields were used for vegetable production in the recent two decades. The base dressing followed the local practice, 5 kg mixed soils were loaded into a pot, watered to 100% field capacity, and stabilized for 1 week before being transplanted. The seeds of the tomato cultivar *Hezuo906* is an anti-mosaic-virus cultivar, which was sterilized, germinated, and nurtured. Tomato seeds were soaked in 0.5% NaClO solution for 20 min, then washed by deionized water twice, and placed in Petri dishes to be germinated in a dark incubation chamber (CWI800, Sheyan, Shanghai, China) at 25°C. The 3-day-germinated seeds were sown into a peat-pearlite mixture (2:1, v/v) in a naturally lit greenhouse and watered with a mixture of 1% urea and 1% potassium phosphate. Twenty-day-old seedlings with four true leaves were transplanted into pots. Soils in pots were watered to 80% field capacity at a frequency of 1 or 2 days. Foliar spray and top dressing were conducted every 10 days six times commencing from the initial fruiting stage. The soils were covered by plastic films when the foliar application was conducted to avoid fulvic acids dropping into the soils. The temperature and light intensity were recorded every 10 min by loggers L95-82 and L99-LX (Hangzhou Loggertech Co., China), respectively. The entire growth period was 126 days from August 31 to January 03 in the same naturally lit greenhouse with an average temperature of 18.7°C ± 3.6°C, a light intensity of 253 ± 212 μmol m^–2^ s^–1^, and daily light integral of 12.6 ± 4.3 μmol m^–2^ day^–1^.

**TABLE 3:** The chemical properties of the experimental soil for Experiment 1.

**pH**	**EC (dS m^–1^)**	**SOM %**	**Available N (μg g^–1^)**	**Available P (μg g^–1^)**	**Available K (μg g^–1^)**	**CEC (cmol kg^–1^)**
7.5	0.47	1.9	342	169	303	19.4

### Experiment 2: Germination of Tomato Seeds

Experiments 2 and 3 were conducted to explore the mechanisms of fulvic acids increasing the production and quality of vegetables. We conducted Experiment 2 to explore the stimulation of fulvic acids on seed germination rate and radicle elongation (Supplementary Figure 2). It was a randomized complete block design with eight concentrations of fulvic acids in solution: 0, 10, 20, 40, 80, 160, 320, and 640 mg L^–1^ with three replicates for each treatment. Two Chinese cultivars, i.e., *Huangmenren* (Jinfa Seed Company, Cangzhou, China) and *Zizhenzhu* (Huashu Seed Company, Qingxian, China) were used, and seeds were sterilized and germinated as in Experiment 1. The temperature and humidity in the incubation chamber were 28°C and 70%, respectively. The seed germination experiment lasted for 7 days. The fulvic acid solution was supplied daily to avoid water deficiency.

### Experiment 3: Hydroponic Experiment With Tomato Plants

We conducted a hydroponic experiment with tomato plants to explore the effects of fulvic acids on root elongation and plant production (Supplementary Figure 3). The experiment was a randomized complete block design with eight concentrations of fulvic acids and four replicates for each treatment. Eight concentrations of fulvic acids in hydroponic nutrient solution were 0, 10, 20, 40, 80, 160, 320, and 640 mg L^–1^.

Tomato seeds of cultivar cv. *Zizhenzhu* were sterilized, germinated, and grown till transplanting as in Experiment 1. Seedlings with four true leaves in similar sizes were selected and then transplanted into containers with 5 L 1/2 Hoagland nutrient solution, and the solutions were shifted to a full-strength nutrient solution at the second week. The macronutrients of Hoagland solution consisted of 4 mM Ca(NO_3_)_2_⋅4H_2_O, 6 mM KNO_3_, 1 mM NH_4_H_2_PO_4_, and 2 mM MgSO_4_⋅7 H_2_O, and the micronutrients were the universal formula (mg L^–1^): 2.86, H_3_BO_3_; 13.9, FeSO_4_⋅7H_2_O; 1.81, MnCl_2_⋅4H_2_O; 0.22, ZnSO_4_⋅7H_2_O; 0.08, CuSO_4_⋅5H_2_O; and 0.02, (NH_4_)_6_Mo_7_O_4_⋅4H_2_O. The nutrient solution’s pH was maintained at about 6.5 by daily adjusting using 0.25 M H_2_SO_4_ or 0.5 M NaOH. All the plants were harvested after growth for 21 days from June 24 to July 15.

### Sampling Methods

The tomato fruits were harvested twice a week once matured and stored in a −20°C fridge. All the fruits were cut finely and homogenized thoroughly after final harvest, using a fruit blender. The homogenates were centrifuged at 3,000 *g* for 10 min and filtered by a syringe filter (0.45 μM) for biological analysis. At the final harvest, the leaves, stems, and roots of tomatoes were separated and collected. Separated tissues were cleaned and green-killed, then dried at 65°C to a constant weight to record the dry mass. Fresh portions were stored in a −20°C fridge till analysis. After plant sampling, the soils in pots were sieved at 1 mm after mixing and air-drying to determine soil chemical properties.

### Measurements of Seed Germination and Radicle Length

We took daily photos of each Petri dish in Experiment 2 with a digital camera (5D Mark IV, Cannon, Germany). The germination rates were achieved by counting the number of germinated seeds in the photos. The radicle length of each seed was measured by ImageJ (Version 1.51a, National Institute of Health, United States). The germination rate and vigor index are calculated using Equations (1) and (2), respectively.


(1) $$G\,e\,r\,m\,i\,n\,a\,t\,i\,o\,n\,r\,a\,t\,e=\frac{N\,u\,m\,b\,e\,r\,o\,f\,g\,e\,r\,m\,i\,n\,a\,t\,e\,d\,s\,e\,e\,d\,s}{T\,o\,t\,a\,l\,n\,u\,m\,b\,e\,r\,o\,f\,s\,e\,e\,d\,s\,s\,o\,w\,n}\times 100\%$$

(2) $$V\,i\,g\,o\,r\,i\,n\,d\,e\,x=\left(m\,e\,a\,n\,r\,a\,d\,i\,c\,l\,e\,l\,e\,n\,g\,t\,h\right)\times \sum \frac{G_{n}}{D_{n}}$$

where *G*_*n*_ is the number of germinated seeds at the *n*th day and *D*_*n*_ is the number of the *n*th day.

### Determinations of Root Morphology

The fresh roots in Experiment 3 were washed by deionized water three times and scanned (V700, Epson, Japan) to obtain root images. The images were then analyzed by WinRhizo Pro (Version 2013, Regent, Canada) to determine root length and the number of root tips.

### Determinations of Soil Property

Soil organic matter (SOM) was determined by dichromate titration. Soil pH was extracted by deionized water at a soil–water ratio of 1:2.5 and determined by a pH meter. Soil available N was determined by the alkali-hydrolyzed reduction diffusing method using 1 M NaOH. Available P was extracted by 0.5 M NaHCO_3_ and determined by the molybdenum-antimony anti-spectrophotometric method. Available K was extracted by 1 M CH_3_COONH_4_ and determined by ammonium acetate extraction-flame photometry (Bao, 2000).

### Determination of Tomato Quality

The total soluble solids in the juice of tomato fruits were analyzed by a portable digital sugar meter (PAL-1, Atago, Japan). The fruit juice was used for the determination of various compounds. The concentrations of C and N in the ground plant tissues were measured by a CNS analyzer (Vario MAX CNS, Elementar, Germany). The concentrations of P, K, Ca, Mg, S, Fe, Mn, Cu, and Zn were determined by an ICP-OES (Optima 8000, PerkinElmer, Germany), using the HNO_3_-H_2_O_2_-digested solutions or the fruit juice of the tomato. The concentrations of heavy metals Cd, Cr, and Pb were measured by an ICP-MS (X Series II, Thermo Fisher, United States). The concentrations of metalloids As and Hg were measured by an X-ray Fluorescence Spectrometer (Axios-Advanced, PANalytical, Netherlands). The quantity and composition of metabolites in tomato fruits were determined by LC-MS (Thermo Vanquish UHPLC, Company Thermo Fisher, United States), coupled with an Orbitrap Q Exactive series mass spectrometer (Orbitrap Q Exactive, Company Thermo Fisher, United States). The fresh tomato fruits (100 mg) were individually ground using a blender and centrifuged and then the homogenate was passed through a 0.45-μM membrane. The supernatant was injected onto an Hyperil Gold column (100 mm × 2.1 mm, 1.9 μm), using a 16-min linear gradient at a flow rate of 0.2 mL min^–1^ for analysis. The Orbitrap Q Exactive series mass spectrometer was operated in positive/negative polarity mode with spray voltage of 3.2 kV, capillary temperature of 320°C, sheath gas flow rate of 35 arb, and aux gas flow rate of 10 arb.

### Statistical Analysis

All the data were analyzed according to the experimental design, using SPSS 22 software (SPSS Statistics 22.0, IBM, United States). Pictures were drawn by Origin 2016 (Origin 2016, OriginLab, United States) and ImageJ (ImageJ 1.8.0, National Institutes of Health, United States). The means of these parameters were compared using Duncan’s multiple range test at *P* < 0.05.

## Results

### Effects of Fulvic Acids on Soil-Culture Tomatoes

**FIGURE 1:**
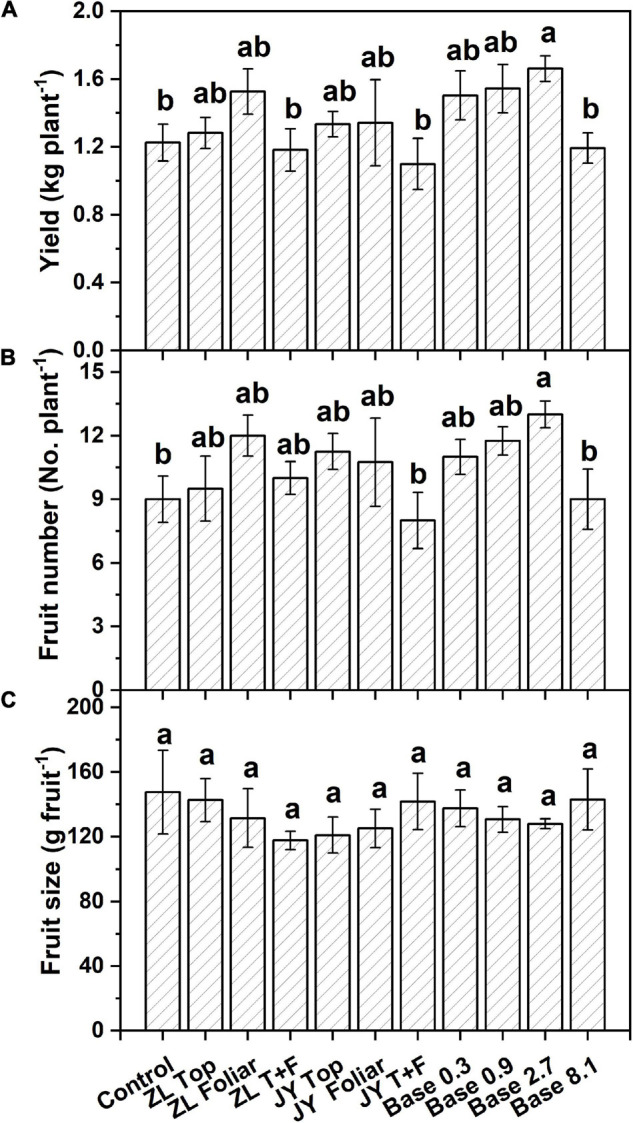
The effect of fulvic acids on total yield **(A)**, fruit number **(B)**, and fruit size **(C)** of tomato plants cv. *Hezuo906* grown in soil culture for 126 days from transplanting at the final harvest. Data are means ± s.e (*N* = 5). The same letters denote insignificant differences (*P* < 0.05) among treatments according to Duncan’s new multiple range test (Experiment 1).

Moderate doses of fulvic acids applied by base and top dressing improved the yield and nutrient quality of tomato fruits in general. More specifically, base dressing of ZL fulvic acids of 2.7 g kg^–1^ increased the yield and fruit number of tomatoes in soil cultures by 35.0 and 44.4% greater than other doses (Figure 1). The other commercial JY fulvic acids with top dressing increased the total biomass of tomato plants by 22.4% (Table 4).

**TABLE 4:** The effect of fulvic acids on plant height, leaf number, and dry matter of stem, leaf, and the entire plants of tomato plants cv. *Hezuo906* grown in the soil culture for 126 days from transplanting (Experiment 1).

	**Plant height (m)**	**Leaf number (No. plant^–1^)**	**Root**	**Stem**	**Leaf**	**Total**	**Root/shoot**
				
			**(g plant^–1^)**				
Control	1.08 a	10.3 c	1.83 bc	20.5 ab	34.5 ab	56.8 ab	0.033 ab
ZL top	1.15 a	12.7 abc	1.85 bc	20.6 ab	30.5 ab	53.0 ab	0.036 ab
ZL foliar	1.15 a	11.3 bc	1.44 bc	15.5 b	26.6 b	43.5 b	0.034 ab
ZL T + F	1.17 a	15.7 a	1.72 bc	20.8 ab	34.6 ab	57.2 ab	0.033 ab
JY top	1.24 a	15.8 a	2.00 ab	26.6 a	40.9 a	69.5 a	0.031 b
JY foliar	1.16 a	15.8 a	1.33 c	18.3 ab	30.0 ab	49.7 b	0.028 b
JY T + F	1.11 a	13.7 abc	1.56 bc	18.2 ab	34.5 ab	54.2 ab	0.031 b
Base 0.3	1.28 a	12.0 abc	1.49 bc	19.8 ab	31.4 ab	52.7 ab	0.029 b
Base 0.9	1.09 a	13.0 abc	1.65 bc	18.7 ab	36.4 ab	56.7 ab	0.031 b
Base 2.7	1.09 a	12.8 abc	1.71 bc	19.4 ab	34.9 ab	56.0 ab	0.032 ab
Base 8.1	1.22 a	14.8 ab	2.38 a	19.9 ab	38.9 a	61.2 ab	0.041 a

*Total biomass is the biomass of entire plants but without fruits. Root/shoot is the ratio of root dry biomass to shoot dry biomass. Data are means (*N* = 5). The same letters denote insignificant differences (*P* < 0.05) among treatments according to Duncan’s new multiple range test.*

**FIGURE 2:**
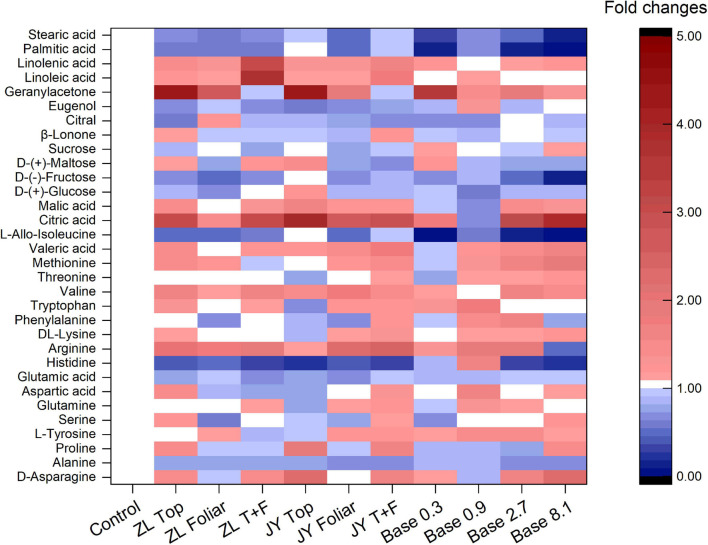
The effect of fulvic acids on the fold changes of quality-related can carbon containing substances in fruit juices of tomato plants grown in soil cultures (Experiment 1).

The base dressing of 0.9 and 2.7 g kg^–1^ tended to increase fold changes of essential and non-essential amino acids (Figure 2). Base dressing of 2.7 g kg^–1^ fulvic acids increased fold changes of phenylalanine, valine, and methionine by 55, 56, and 61%, respectively. Compared with the control, ZL fulvic acids by top and foliar application increased the fold changes of linolenic and linoleic acid by 209 and 275%, respectively. Fulvic acids increased fold changes of citric and malic acid with the greatest increased by 291 and 67% in JY top dressing treatment, 211 and 42% in Base 2.7 (Figure 2). However, fulvic acids in all treatments did not affect fold changes of soluble sugar (glucose, fructose, maltose, and sucrose) and some aromatic substances (β-lonone, citral, and eugenol) (Figure 2).

The base dressing of 2.7 g kg^–1^ also increased the concentrations of mineral elements in tomato juice, especially Mg by 55.9%, Ca by 31.4%, and Zn by 43.1% (Table 5). The concentrations of Mg, S, Ca, and Fe increased in all modes of application.

**TABLE 5:** The effect of fulvic acids on mineral concentration (μg g^–1^) in fruit juices of tomato plants cv. *Hezuo906* grown in the soil culture for 126 days from transplanting (Experiment 1).

	**K**	**P**	**Mg**	**S**	**Ca**	**Fe**	**Zn**	**Mn**	**Cu**
Control	1755 bc	144.8 ab	38.3 c	37.6 a	11.8 bc	0.30 d	0.320 c	0.110 d	0.095 d
ZL top	2049 ab	152.4 ab	54.3 ab	45.9 a	13.3 bc	0.66 ab	0.480 ab	0.203 ab	0.143 abc
ZL foliar	1789 bc	135.8 ab	53.9 ab	43.7 a	15.0 abc	0.54 bcd	0.445 bc	0.175 bcd	0.136 abcd
ZL T + F	1520 c	98.4 b	63.4 a	44.7 a	16.0 abc	0.60 abc	0.540 ab	0.213 ab	0.129 cd
JY top	1889 bc	119.6 ab	54.0 ab	43.8 a	20.5 a	0.52 bcd	0.533 ab	0.178 bcd	0.123 cd
JY foliar	1597 c	104.7 b	47.7 bc	40.5 a	12.5 bc	0.44 cd	0.410 bc	0.147 cd	0.114 cd
JY T + F	2196 a	179.3 a	59.5 a	48.2 a	13.4 bc	0.67 ab	0.635 a	0.253 a	0.163 a
Base 0.3	1712 bc	117.9 ab	36.9 c	39.0 a	9.7 c	0.35 cd	0.350 c	0.128 d	0.098 d
Base 0.9	1899 bc	149.8 ab	46.8 bc	41.0 a	11.3 bc	0.40 cd	0.430 bc	0.138 d	0.125 cd
Base 2.7	2064 ab	136.0 ab	50.9 abc	40.5 a	15.5 abc	0.47 bcd	0.458 abc	0.152 cd	0.134 abc
Base 8.1	2251 a	165.7 ab	59.7 a	45.7 a	17.9 ab	0.80 a	0.623 a	0.197 abc	0.157 ab

*Data are means (*N* = 5). The same letters denote insignificant differences (*P* < 0.05) among treatments according to Duncan’s new multiple range test.*

Fulvic acids by top and base dressing tended to decrease soil pH and increase the soil CEC and SOM across all treatments while foliar application did not affect these soil properties (Table 6). More specifically, Base 2.7 treatment decreased soil pH from 8.06 to 7.81 while it increased EC by 102.9%.

**TABLE 6:** The effect of fulvic acids on the properties of soils where tomato plants cv. *Hezuo906* were grown for 126 days from transplanting (Experiment 1).

	**pH**	**EC (dS m^–1^)**	**SOM (%)**	**Available N (μg g^–1^)**	**Available P (μg g^–1^)**	**Available K (μg g^–1^)**	**CEC (cmol kg^–1^)**
Control	8.06 ab	0.34 cd	1.87 c	106 b	151 a	110 b	18.9 b
ZL top	7.81 de	0.52 bc	1.91 bc	114 b	152 a	108 b	19.2 ab
ZL foliar	8.14 a	0.30 d	1.88 c	110 b	154 a	112 b	19.2 ab
ZL T + F	7.90 cd	0.45 cd	1.90 bc	113 b	144 a	115 ab	19.4 ab
JY top	7.75 de	0.57 bc	1.93 bc	113 b	152 a	107 b	19.4 ab
JY foliar	8.02 abc	0.36 cd	1.88 c	108 b	154 a	103 b	19.1 ab
JY T + F	7.74 e	0.50 bcd	1.91 bc	111 b	144 a	116 ab	19.4 ab
Base 0.3	8.05 abc	0.35 cd	1.92 bc	110 b	149 a	102 b	19.3 ab
Base 0.9	8.00 bc	0.41 cd	1.94 bc	107 b	151 a	108 b	19.6 ab
Base 2.7	7.81 de	0.69 b	1.96 ab	116 b	148 a	105 b	19.5 ab
Base 8.1	7.46 f	1.68 a	2.01 a	161 a	144 a	136 a	19.8 a

*Data are means (*N* = 5). The same letters denote insignificant differences (*P* < 0.05) among treatments according to Duncan’s new multiple range test. EC, electrical conductance; SOM, soil organic matter; CEC, cation exchange capacity.*

### Effects of Fulvic Acids on Germination of Tomato Seeds

**FIGURE 3:**
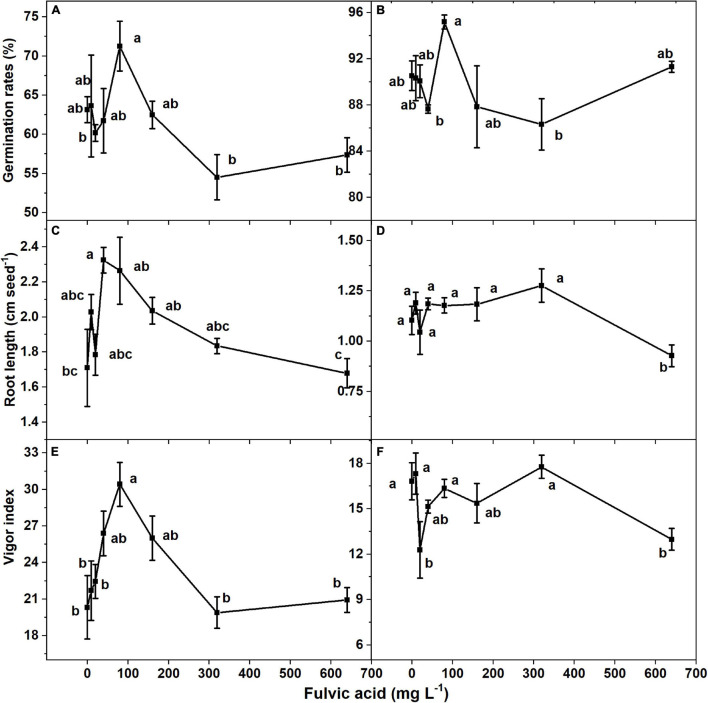
The effect of fulvic acids on the germination rate, root length, and vigor index in of tomato seeds cv. *Huangmeiren* **(A,C,E)** and cv. *Zizhenzhu* **(B,D,F)** germinated for 7 days. Data are means ± s.e (*N* = 3). The same letters denote insignificant differences (*P* < 0.05) among treatments according to Duncan’s new multiple range test (Experiment 2).

The seed germination rates of both tomato cultivars increased and then decreased when the concentration of fulvic acids increased after 7 days (Figures 3A,B). Specifically, the most effective seed germination rate was 12.9% greater than the control when fulvic acids were at a concentration of 80 mg L^–1^ on average. Fulvic acids of 80 mg L^–1^ also increased the radicle length and vigor index by 32.2 and 49.7% compared with the control, respectively (Figure 3). The promotion of seed germination by fulvic acids on tomato seed cv. *Huangmeiren* was greater than that of cv. *Zizhenzhu*. The average seed germination rates of *Huangmeiren* and *Zizhenzhu* across the various concentrations of fulvic acids were 62.6 and 89.9%, respectively.

### Effects of Fulvic Acids on Hydroponic Tomatoes

**FIGURE 4:**
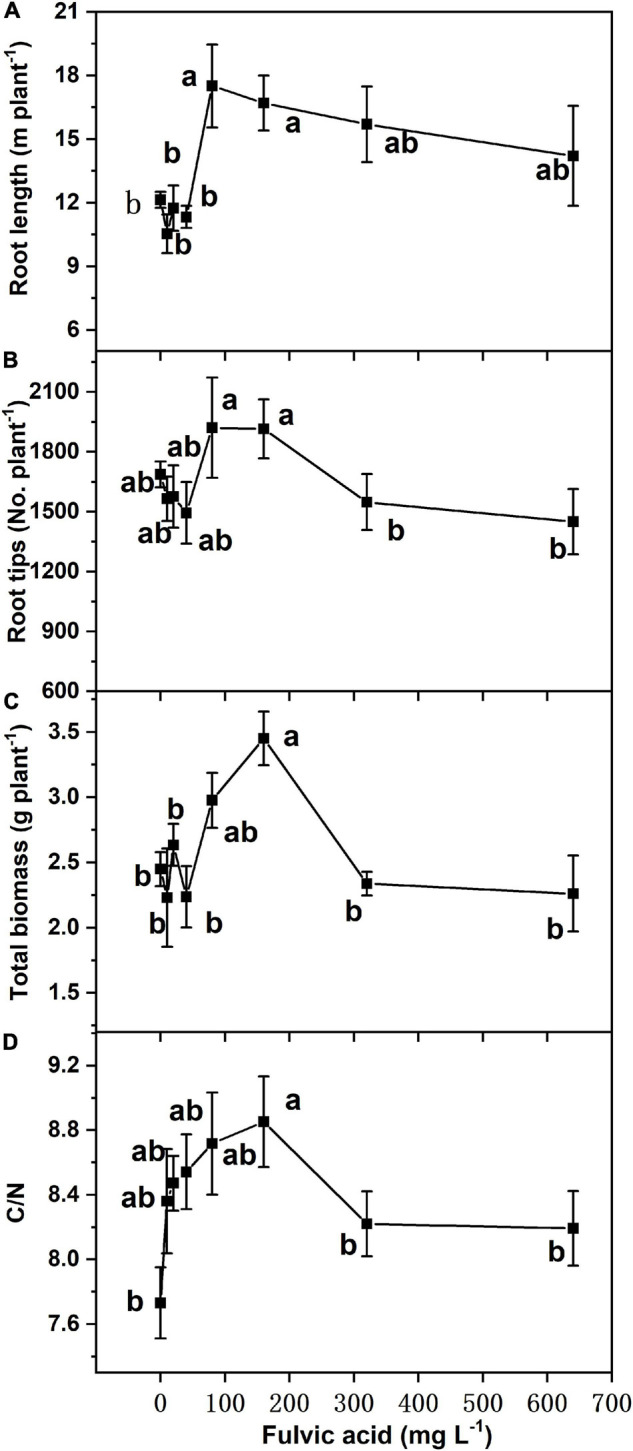
The effect of fulvic acids on root length **(A)**, root tips **(B)**, total biomass **(C)**, and ratio of carbon to nitrogen **(D)** in leaves of tomato plants cv. *Zizhenzhu* grown in hydroponics for 21 days. Data are means ± s.e (*N* = 4). The same letters denote insignificant differences (*P* < 0.05) among treatments according to Duncan’s new multiple range test (Experiment 3).

Similar to seed germination, the effects on total biomass, foliar *C*/*N* ratio, and root growth of tomato plants in hydroponics increased at first and then decreased when the fulvic acid concentrations increased (Table 7 and Figure 4). Fulvic acids increased the total biomass and foliar C/N ratio by 40.8 and 14.5% at concentrations of 160 mg L^–1^, respectively, compared with the control. Fulvic acids increased the root length and number of lateral root tips of hydroponic tomato plants (Figure 4). The optimal concentration of fulvic acids on root growth was 80 mg L^–1^, which increased root length and root tips by 44.4 and 13.8% when compared with the control, respectively (Figure 4 and Supplementary Figure 4). Fulvic acids of 80 mg L^–1^ also increased the concentrations of mineral elements in the leaf, especially Fe, by 109% compared with the control (Table 7).

**TABLE 7:** The effect of fulvic acids (FA) on nutrient concentration in leaves of tomato plants cv. *Zizhenzhu* in hydroponics for 21 days from transplanting (Experiment 3).

**FA (mg L^–1^)**	**P**	**K**	**Ca**	**Mg**	**S**	**Fe**	**Mn**	**Zn**
		
					**(μg g^–1^)**			
0	45.5 a	339 a	143 a	33.8 a	56.2 abc	2.46 b	1.18 a	0.435 ab
10	41.8 a	287 ab	138 a	29.1 a	50.1 abc	1.51 b	0.83 a	0.411 ab
20	42.3 a	262 b	143 a	32.7 a	44.4 c	1.66 b	0.73 a	0.387 b
40	40.3 a	279 b	136 a	33.1 a	46.1 bc	1.48 b	0.88 a	0.447 ab
80	43.8 a	289 ab	151 a	32.3 a	49.7 abc	9.43 a	1.17 a	0.440 ab
160	41.3 a	287 ab	138 a	32.4 a	48.4 bc	1.85 b	1.09 a	0.561 a
320	43.7 a	296 ab	141 a	29.6 a	57.0 ab	2.69 b	1.10 a	0.499 ab
640	42.0 a	280 b	133 a	27.7 a	61.3 a	3.66 b	0.92 a	0.484 ab

*Data are means (*N* = 4). The same letters denote insignificant differences (*P* < 0.05) among treatments according to Duncan’s new multiple range test.*

Another soil-culture experiment with pak choi was conducted to expand the application of fulvic acids on leafy vegetables with shallow roots using the soils in Experiment 1 and a series of concentrations of fulvic acids the same as Experiments 2 and 3 (Supplementary Figure 5). The aboveground portions of pak choi were harvested on the 21st and 30th day from seeding, respectively. The yield and mineral concentrations in the edible portion were measured (Supplementary Table 1 and Supplementary Figure 6). The analysis of the cost and benefit of fulvic acid application is calculated in Supplementary Table 2. Results are shown in the Supplementary Material.

## Discussion

Humic substances are the end products of microbial decomposition derived from plant residuals, which play key roles in various soils and influence plant functions. Fulvic acids are components of humic substances according to their solubility and molecular weight. There are similarities between humic substances and fulvic acids, but the effects on plants depend on the source organic matter, the plant species, and the growth medium (Calvo et al., 2014). Although the effects of humic-like fertilizers differ, studies confirm their effectiveness and economic value in agricultural production. However, it lacks studies focusing on the effects of renewable sources of fulvic acids on vegetable production and yield under various growth mediums and the underlying mechanisms. Our study investigates the effects of straw-derived fulvic acids on the entire growth period of tomato from seed to fruit under either hydroponic or soil culture and give certain explanations on how fulvic acids work.

### Fulvic Acids Increased the Yield and Production of Vegetables

Fulvic acids at moderate concentrations and applications of base and top dressing increased the production and yield of tomato plants in our study (Table 4, Figures 1, 4 and Supplementary Figure 6). The yield increments of tomato and pak choi can reach 35.0 and 54% (Figure 1 and Supplementary Figure 6) while fulvic acids increased the total biomass of hydroponic tomato plants by 40.8% at most (Figure 4C). The improved yield could contribute to the farmer’s income to an extent (Supplementary Table 2). Similarly, humic-like substances were reported to increase the production of various vegetable plants. Atiyeh et al. (2002) report that humic substances extracted from vermicompost of pig manures and food waste increased the shoot and root dry masses of tomato seedlings with the optimal concentration of the humic substance being 0.2–0.5 g kg^–1^. Karakurt et al. (2009) treated 20 ml L^–1^ humic-like substances with soil and increased the early and final yield of peppers by 38.3 and 11.8% compared with the control. The optimal doses in Karakurt et al. (2009) were lower than our current study; however, the fulvic acids in our study increased the plant yield to a greater extent. Our study demonstrates that fulvic acids have little effect on fruit size (Figure 1C), different from previous studies (Ferrara and Brunetti, 2010; Naidu et al., 2013; Suh et al., 2014). These differences in our study could be attributed to the variety of crops and the methods of cultivation, and the extent of increments and the specific performances also differed from the species of the plants and the sources of the humic-like substances (Morard et al., 2010). Haghighi et al. (2012) conclude that the mechanism of the increase in yield was the stimulation on N metabolism and photosynthesis activity, and Anjum et al. (2011) record that the application of fulvic acids increased the net photosynthesis, transpiration rate, and intercellular concentration of CO_2_—effects that were related to plant growth promotion.

### Fulvic Acids Increased the Quality of Vegetables

In our study, fulvic acids increased the concentrations of minerals and carbon-containing substances related to product quality in the edible portion of vegetables (Table 7, Supplementary Table 1, Figure 2, and Supplementary Figure 6). Several studies confirm an improvement of vegetable quality by humic-like substance application (Haghighi and Teixeira Da Silva, 2013). Fulvic acids are demonstrated to improve the produce quality, e.g., by inducing the accumulation of secondary metabolites, vitamins, antioxidants, and minerals (Calvo et al., 2014; Gruda et al., 2018), confirming our results.

More specifically, fulvic acids increase the concentrations of minerals at moderate doses, especially Mg, Fe, and Zn in either tomato or pak choi (Table 5 and Supplementary Table 1). It is demonstrated that the application of humic acids enhances the accumulation of minerals such as Ca, Fe, Mg, and Zn in soil-cultured garlic (Denre et al., 2014). Similarly, humic substances increased the concentrations of P and Fe in grapes (Sánchez-Sánchez et al., 2006). With respect to carbon-containing substances, base dressing of 2.7 g kg^–1^ fulvic acids increased fold changes of phenylalanine, valine, and methionine, which possibly improved the fragrance of tomato berries (Ou et al., 2007), and the increased arginine contributed to an improvement in the quality of fruit berries (Micallef and Shelp, 1989; Nasibi et al., 2011). Consistent with our study, humic acids increased the concentrations of linolenic and linoleic acid in rapeseed (Amiri et al., 2020), the titratable acids in citrus (Hameed et al., 2018), and the amino acids in tomatoes (Yildirim, 2007). However, fulvic acids did not affect fold changes of soluble sugar and some aromatic substances (Figure 2), similar to a previous study (Canellas et al., 2013). That could be attributed to the application of fulvic acids that decreased the activity of enzymes involved in glucose metabolism (Canellas et al., 2015). The metabolites alternatively could be used to increase the growth of plants (Canellas et al., 2013), which confirms our results of tomato production and yield (Table 4 and Figures 1, 2). According to Trevisan et al. (2011), Jannin et al. (2012), and Canellas et al. (2015), the effects of fulvic acids on nutrients may be attributed to the regulation of enzymes and genes that are involved in the primary metabolism, and thus affect the transformation and accumulation of metabolic substances.

The increased quality indicators in tomato fruits by fulvic acids indicates an improved fruit quality and, thus, human nutrition in general. However, the quality of vegetables was not always improved by fulvic acids, such as some reduced sugars in tomato fruits (Figure 2) and mineral concentration in the edible portion of pak choi (Supplementary Table 1), so one need to be cautious when applying. These findings suggest that plant bio-stimulants, i.e., fulvic acids, may be used to improve product quality of vegetables in a sustainable way (Gruda et al., 2018). This will be imperative in the near future due to projected climate changes with high temperatures, water scarcity (Gruda et al., 2019a, b), and high CO_2_ concentrations (Dong et al., 2018, 2020).

### The Reasons for Increased Yield and Quality

The increases in production and quality might be attributed to the stimulants on plant growth, especially the promotion of root growth and, thus, the uptake of minerals. In our study, fulvic acids increased root growth of germinated seeds (Figure 3) and increased the root length and number of lateral root tips of hydroponic tomato plants (Figures 4A,B) while the uptake of mineral nutrients and the plant biomass increased (Table 7 and Figure 4). Fulvic acids are demonstrated to increase the root length of wheat seeds (Qin et al., 2016) and tomato plants (Dobbss et al., 2007, 2010) although humic extracts from hydrochar and Amazonian Dark Earth increased radicles and seminal lateral roots of maize seeds (Bento et al., 2020). The effects on root growth could be attributed to the hormone-like molecules and their auxin- and gibberellin-like effects that can promote the elongation of root cells, coleoptiles, and hypocotyls (Zhao and Zhong, 2013; Canellas et al., 2015), confirming the characteristics of our microbially fermented fulvic acids. According to Blomster et al. (2011), fulvic acids enhance the activity of auxin signaling and the involved enzymes to increase the growth of roots. However, the current study lacks specific explanation; the roles and mechanisms by which humic substances affect plant growth need further investigation.

The increased root growth was observed to increase the nutrient uptake of vegetable plants. Fulvic acids increased the concentrations of Ca, Fe, and Zn in leaves in hydroponic tomato plants, especially Fe by 109% at most (Table 7). Similar to the effects on Fe in our study, humic acid improved the utilization efficiency of Fe in both the whole plant and roots of tomato seedlings in hydroponics by 46 and 161%, respectively (Adani et al., 1998) while several studies report that fulvic acids can promote the absorption of Fe (Pinton et al., 1997, 1999; Halim et al., 2003; Cerozi, 2020). The effects on the concentrations of Fe could be attributed to the regulation of gene expression, which is related to reduction and transport of Fe, thus improving the iron chelation and availability and, thus, the root uptake (Bocanegra et al., 2006; Elena et al., 2009). In addition, similarly to natural chelators, fulvic acids chelate Fe and other micronutrients and move them through membranes, thus enhancing the mineral accumulation in plants (Calvo et al., 2014).

According to Calvo et al. (2014), humic substances stimulate the growth of root and chelate ions and, thus, increase plant uptake of nutrients. Humic substances upregulate the activities of genes and enzymes involved in the root-to-shoot translocation of nutrients (Mora et al., 2010). Better root growth facilitates the uptake of more nutrients due to the greater surface area. On the other hand, the effect can be attributed to the acidity of fulvic acids (Table 1), which decreases the pH of growth medium and, thus, improves the bioavailability of nutrients (Muscolo et al., 2007). Humic substances are reported to increase the root length and diameter of tomato seedlings and the yield of greenhouse-cultured tomato (Qin and Leskovar, 2020), and they also conclude that the increments were attributed to improvement on the structure of roots. The increase in nutrient uptake further contributed to the promotion on biomass, thus increasing the yield and quality. The *C*/*N* ratio of hydroponic plants shared the similar trends with root growth (Figure 4), which indicated the accumulation of carbohydrate contributing to plant yield and similar to a previous study (Aminifard et al., 2012).

### Fulvic Acids Increased the Germination of Seeds

Similar to the root growth of plants, fulvic acids at moderate concentrations increased the germination rate and vigor index of tomato seeds (Figure 3). Several other studies confirm our results that fulvic acids increase the germination of seeds. Soluble humates extracted from vermicomposted cattle manure in the soil substrate increased the germination rate of tomato seeds by 31.6% (Olivares et al., 2015). Humic substances increased the germination rate and vigor index of cucumber seeds (Ahmed and Awad, 2020). Compared with our study, the humates in Olivares et al. (2015) increased the germination rate of tomato seeds to a greater extent, which might be attributed to their combined application of plant growth–promoting bacterium. The promotion on seed germination might be attributed to the auxin-like substances in humic products that can increase the activity of amylase and promote seed respiration (Zandonadi et al., 2007; Canellas et al., 2010; Canellas and Olivares, 2014). The different performances of seed germination between *Huangmeiren* and *Zizhenzhu* also indicate that fulvic acids facilitate the germination of cultivar with greater germination rates.

### Fulvic Acids Affected the Chemical Properties of Soil

Fulvic acids by top and base dressing tended to decrease soil pH and increase the soil CEC and SOM across all treatments while foliar application did not affect these soil properties (Table 6). Base dressing of 8.1 g kg^–1^ decreased soil pH while increasing soil EC, SOM, concentrations of available N, available K, and CEC to the greatest extent. The decrease in soil pH by fulvic acid application could be attributed to the acidity from fulvic acids (Sharif et al., 2002; Tahir et al., 2011). According to Suntari et al. (2015), fulvic acids increase soil fertility indicated by the increased concentration of available N. However, fulvic acids did not affect soil available P in our study, inconsistent with other reports finding that humic substances could enhance this parameter (Cimrin and Yilmaz, 2005; Jones et al., 2007). We believe that the result of our study can be attributed to the high level of P in the experimental soils (Table 2).

## Conclusion

Our study demonstrates that the moderate application of bioresource compounds, i.e., straw-extracted and microbially fermented fulvic acids, enhanced seed germination, production and yield of vegetables, and vegetable quality to an extent in both hydroponics and soil cultures. The improvement in the growth and quality of vegetables can be attributed to the promotion of root elongation and, thus, increased nutrient uptake by more likely the auxin-like substances. Also, fulvic acids can improve soil fertility indicated by the increased SOM and soil CEC. Our study confirms that optimal concentrations of fulvic acids were 2.7 g kg^–1^ as base dressing and 80–160 mg L^–1^ in solutions as seed soaking, top dressing, and hydroponic application. Future studies should aim to specify the effective components of fulvic acids and to explore the underlying mechanisms of how fulvic acids work from molecular perspectives.

## Data Availability Statement

The original contributions presented in the study are included in the article/Supplementary Material, further inquiries can be directed to the corresponding authors.

## Author Contributions

PZ: conceptualization, methodology, software, and writing—original draft. GW and ZD: resources. XC and PZ: investigation and formal analysis. HZ: investigation and resources. JD: conceptualization, data curation, and writing—review and editing. NG and XL: writing—review and editing. ZD: supervision and project administration. All authors contributed to the article and approved the submitted version.

## Conflict of Interest

The authors declare that the research was conducted in the absence of any commercial or financial relationships that could be construed as a potential conflict of interest.

## Publisher’s Note

All claims expressed in this article are solely those of the authors and do not necessarily represent those of their affiliated organizations, or those of the publisher, the editors and the reviewers. Any product that may be evaluated in this article, or claim that may be made by its manufacturer, is not guaranteed or endorsed by the publisher.
