# Supplementary material for: Dose-Dependent Application of Straw-Derived Fulvic Acid on Yield and Quality of Tomato Plants Grown in a Greenhouse
Source: Front Plant Sci. 2021 Oct 11;12:736613. doi: 10.3389/fpls.2021.736613 (PMC8542715; doi:10.3389/fpls.2021.736613)
Supplement: Supplementary file 1 [file Data_Sheet_1.docx]

Supplementary information

Journal: Frontiers in Plant Science

Date of submission: 18 Aug 2021

Word counts: 4, 990

Number of tables: 7

Number of figures: 4 (colour online-only)

Number of supplementary tables: 2 (colour online-only)

Number of supplementary figures: 6 (colour online-only)

Dose-Dependent Application of Straw-Derived Fulvic Acid on Yield and Quality of Tomato Plants Grown in a Greenhouse

Peijia Zhang ^1, 2^, Hongjia Zhang ^3^, Guoqing Wu ^3^, Xiaoyuan Chen ^3^, Nazim Gruda ^4^, Xun Li ^1^, Jinlong Dong ^1, *^, Zengqiang Duan ^1, *^

^1^ State Key Laboratory of Soil and Sustainable Agriculture, Institute of Soil Science, Chinese Academy of Sciences, Nanjing, China

^2^ University of Chinese Academy of Sciences, Beijing, China

^3^ Nutrition and Health Research Institute, COFCO, Beijing, China

^4^ Institute of Crop Science and Resource Conservation, Division of Horticultural Sciences, University of Bonn, Bonn, Germany

^*^ Correspondence

Running title: Fulvic Acids: Biostimulants of Tomato

Correspondence:

Jinlong Dong

Address: 71 East Beijing Road, Xuanwu District, Nanjing, Jiangsu, China

Email: [jldong@issas.ac.cn](mailto:jldong@issas.ac.cn); Telephone: +86 025 86881577

ORCID: 0000-0002-7766-4409

Zengqiang Duan

Address: 71 East Beijing Road, Xuanwu District, Nanjing, Jiangsu, China

Email: [zqduan@issas.ac.cn](mailto:zqduan@issas.ac.cn); Telephone: +86 025 86881562

TABLE S1. The effect of fulvic acids (FA) on mineral concentration in edible portion of pakchoi plants cv. *Gailiangjinpin28* grown in the soil culture for 33 days.

| FA  (mg L^-1^) | P | K | Ca | Mg |  | | Fe | | Mn | | Cu | Zn | |  |
| --- | --- | --- | --- | --- | --- | --- | --- | --- | --- | --- | --- | --- | --- | --- |
|  | (g kg^-1^) | | | | |  | |  | | (µg g^-1^) | | |  | |
| 0 | 10.7 abc | 60.6 a | 39.40 a | 5.36 a |  | | 219.9 a | | 101.5 a | | 9.98 a | 63.7 bc | |  |
| 10 | 11.4 abc | 54.7 a | 41.54 a | 5.24 ab |  | | 225.7 a | | 117.3 a | | 9.91 a | 61.6 c | |  |
| 20 | 12.3 a | 45.1 a | 44.88 a | 5.14 ab |  | | 208.6 a | | 123.8 a | | 10.34 a | 58.9 c | |  |
| 40 | 12.2 a | 41.6 a | 44.97 a | 5.04 ab |  | | 246.7 a | | 123.1 a | | 10.49 a | 58.4 c | |  |
| 80 | 12.5 a | 49.2 a | 45.31 a | 4.91 b |  | | 176.5 a | | 96.9 a | | 10.18 a | 67.6 bc | |  |
| 160 | 11.7 ab | 45.7 a | 46.59 a | 4.89 b |  | | 206.3 a | | 109.8 a | | 10.51 a | 68.4 bc | |  |
| 320 | 9.8 bc | 65.3 a | 39.01 a | 5.13 ab |  | | 194.5 a | | 113.7 a | | 10.52 a | 74.6 ab | |  |
| 640 | 9.3 c | 65.0 a | 39.80 a | 5.24 ab |  | | 173.2 a | | 124.4 a | | 10.83 a | 82.3 a | |  |

Data are means (*N=4*). The same letters denote insignificant differences (*P*<0.05) among treatments according to Duncan’s new multiple range test.

TABLE S2. The analysis of economic efficiency in unit of US dollars per ha of the use of fulvic acids by base dressing of 2.7 g kg^-1^ (Practice A) and top dressing of 100 mg L^-1^ (Practice B) for six times on tomato plants grown in soils in this study.

| Practice | Additional cost | Yield benefit | Net income |
| --- | --- | --- | --- |
| Practice A | 3038 | 19350 | 16313 |
| Practice B | 2025 | 2250 | 225 |

The calculation was based on the price in China where the prices of fulvic acids and tomato fruits were 2.5 dollars kg^-1^ and 1 dollar kg^-1^. The density of tomato plants was 45000 plants ha^-1^.


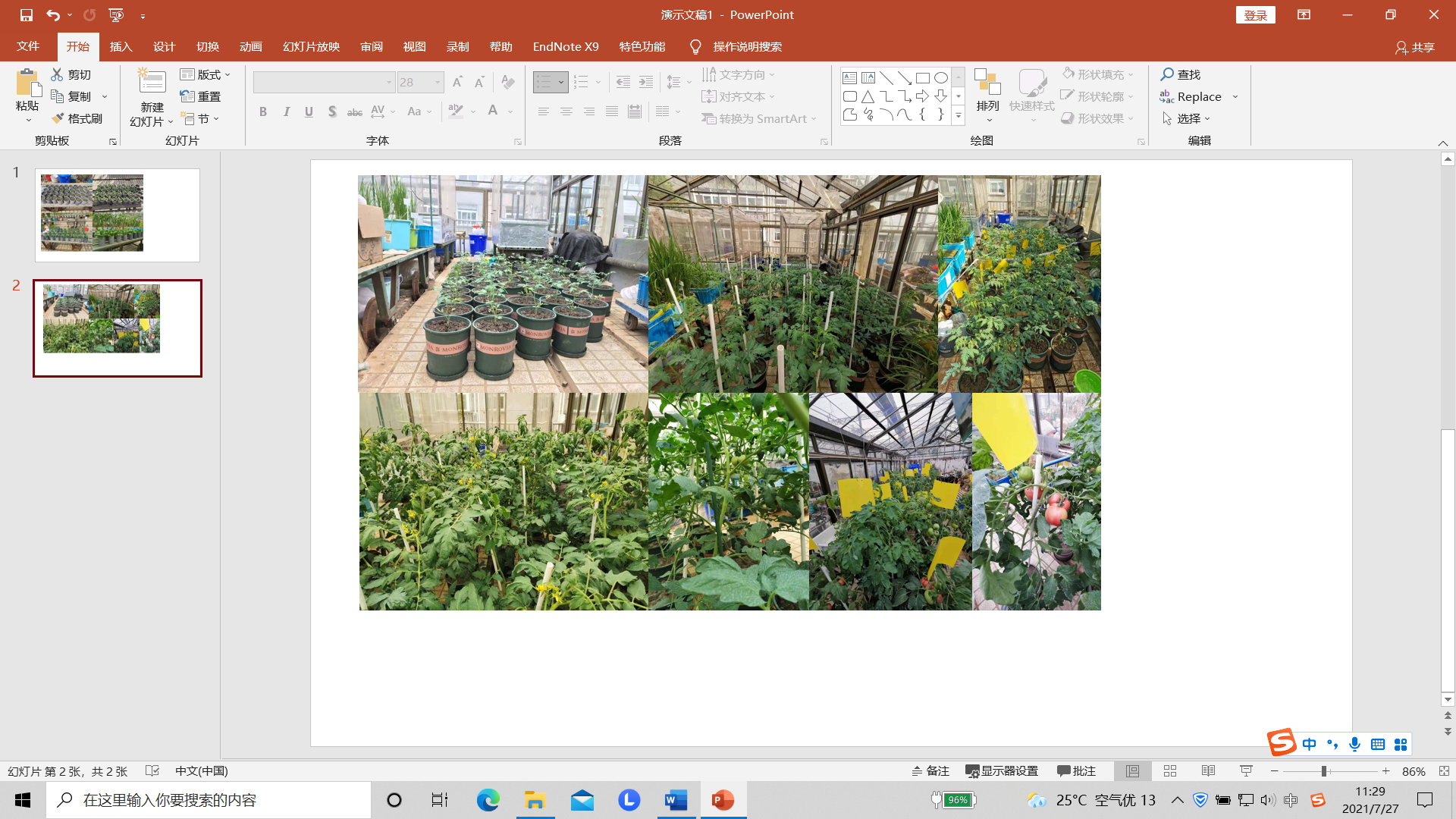
FIGURE S1. The plant growth vigor of tomato plants cv. *Hezuo906* grown in soil culture from transplanting to the fruiting stage (Experiment 1).

FIGURE S2. The photos of the effect of fulvic acids on the seed germination and root length of tomato plants cv. *Zizhenzhu* for an example germinated for 7 days (Experiment 2).


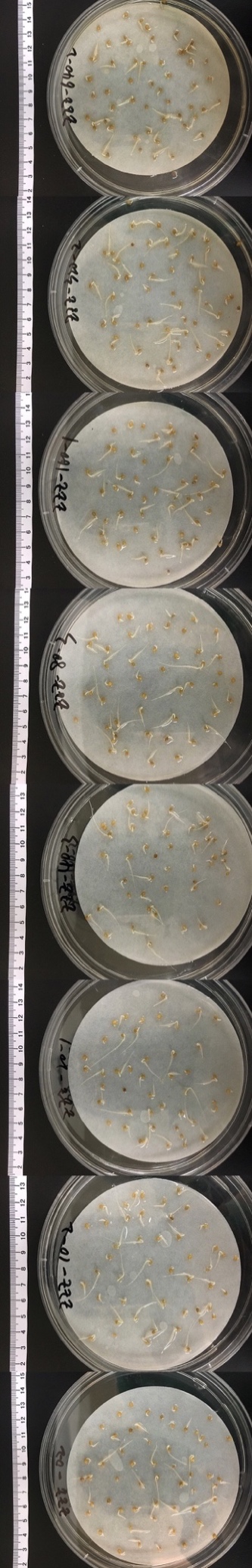


**(a) 0 mg l^-1^**

**(b) 10 mg l^-1^**


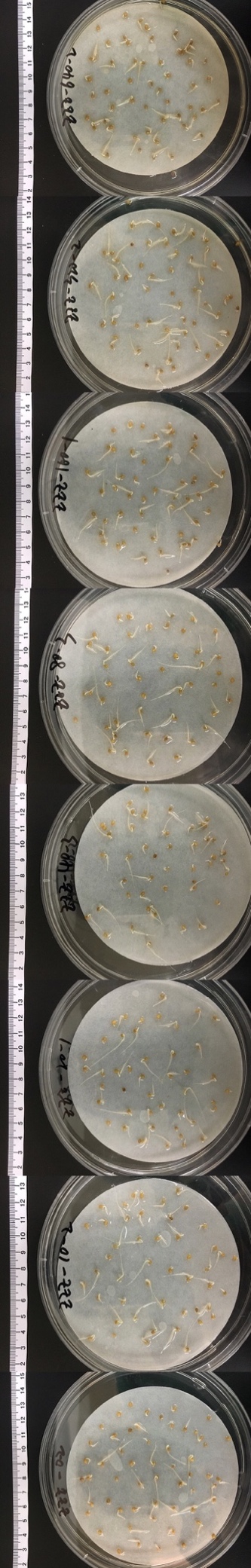


**(c) 20 mg l^-1^**

**(d) 40 mg l^-1^**


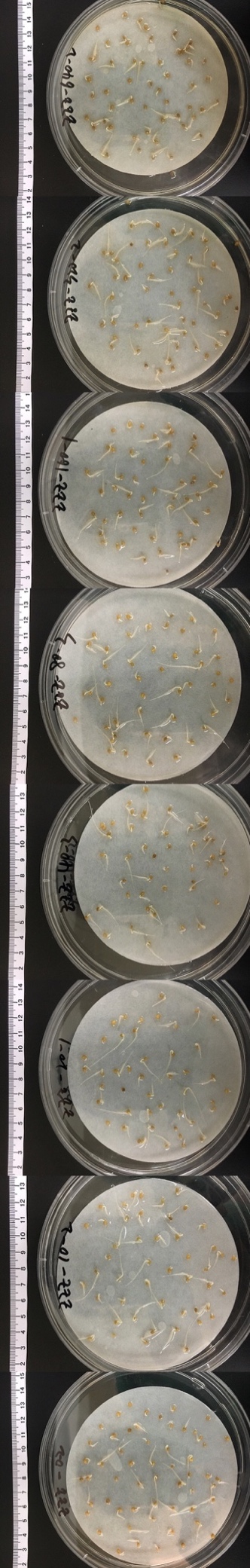


**(e) 80 mg l^-1^**

**(f) 160 mg l^-1^**


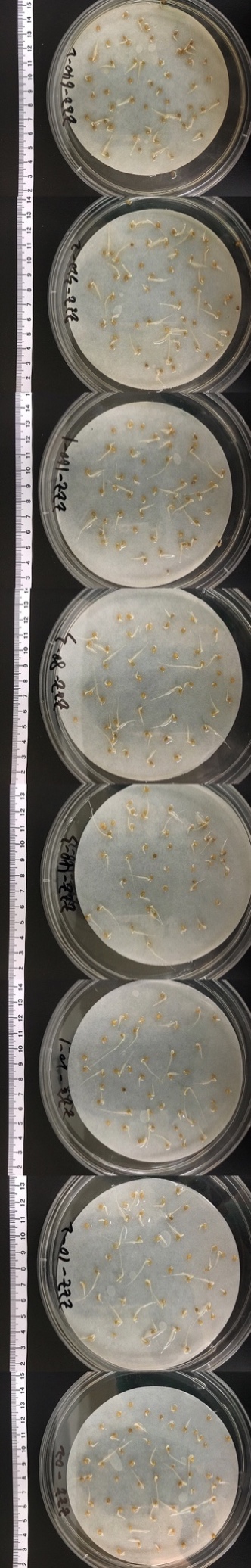


**(g) 320 mg l^-1^**

**(h) 640 mg l^-1^**

FIGURE S3. The growth of tomato plants cv. *Zizhenzhu* grown in hydroponics for 21 days from transplanting (Experiment 3).


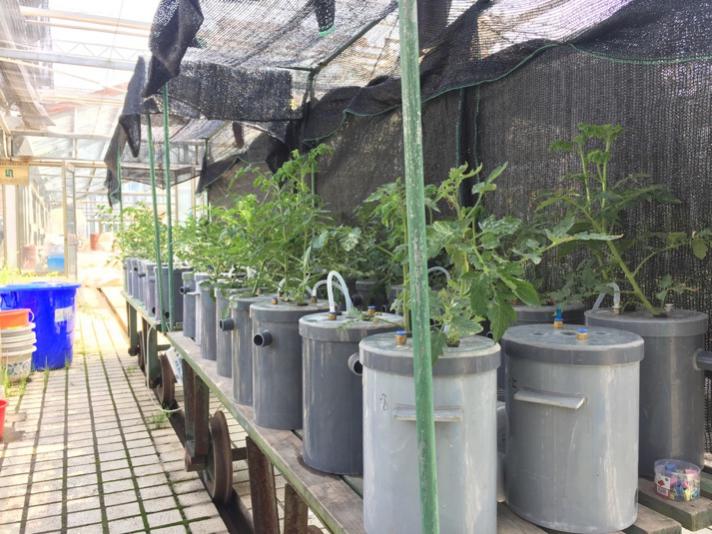

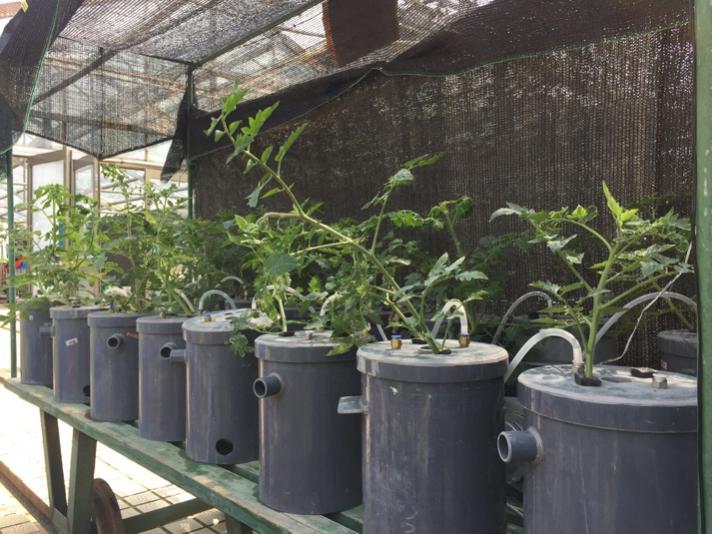


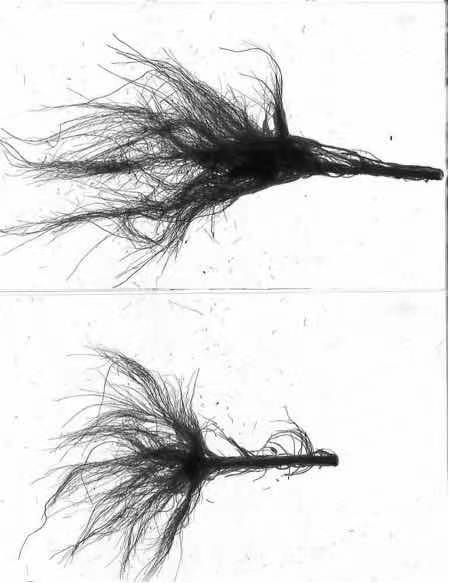


FIGURE S4 The effect of 80 mg L^-1^ fulvic acids (left) on root growth of tomato plants, compared with control (right) (Experiment 3).

FIGURE S5. The growth of pakchoi plants cv. *Gailiangjinpin28* grown in soil cultures for 15 (a) and 33 days (b) from seeding.


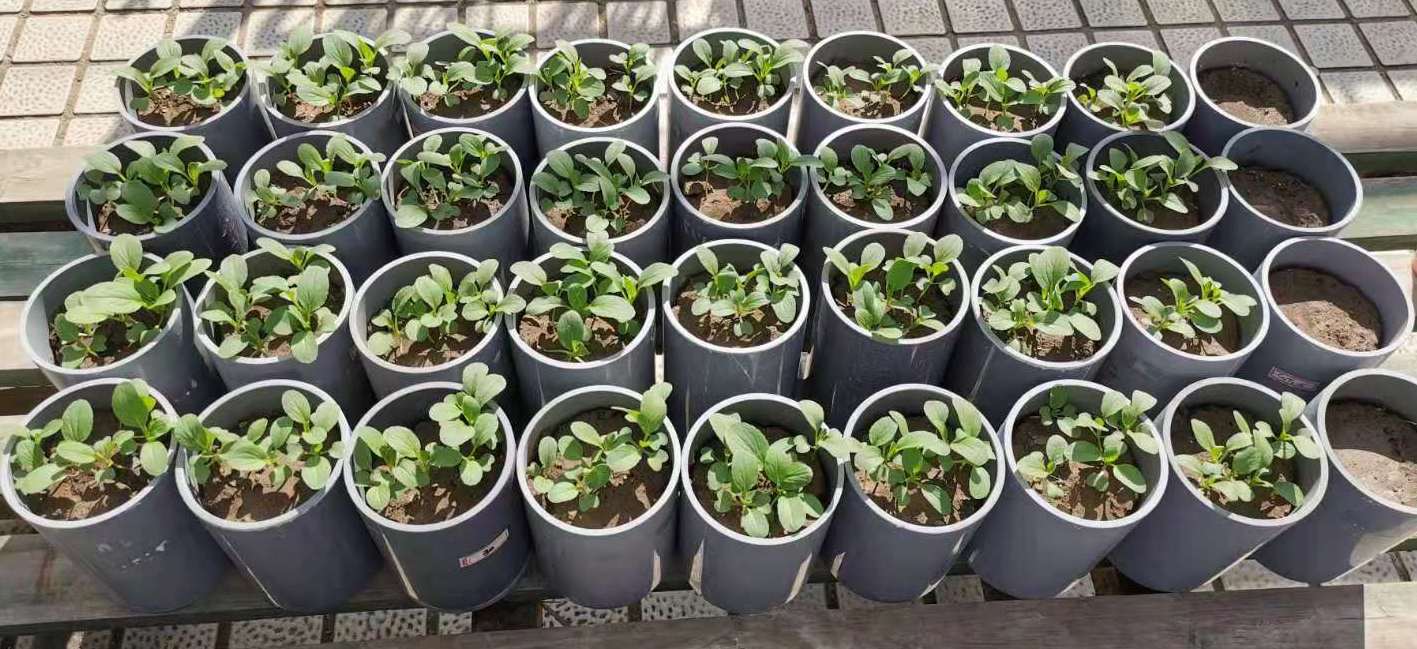

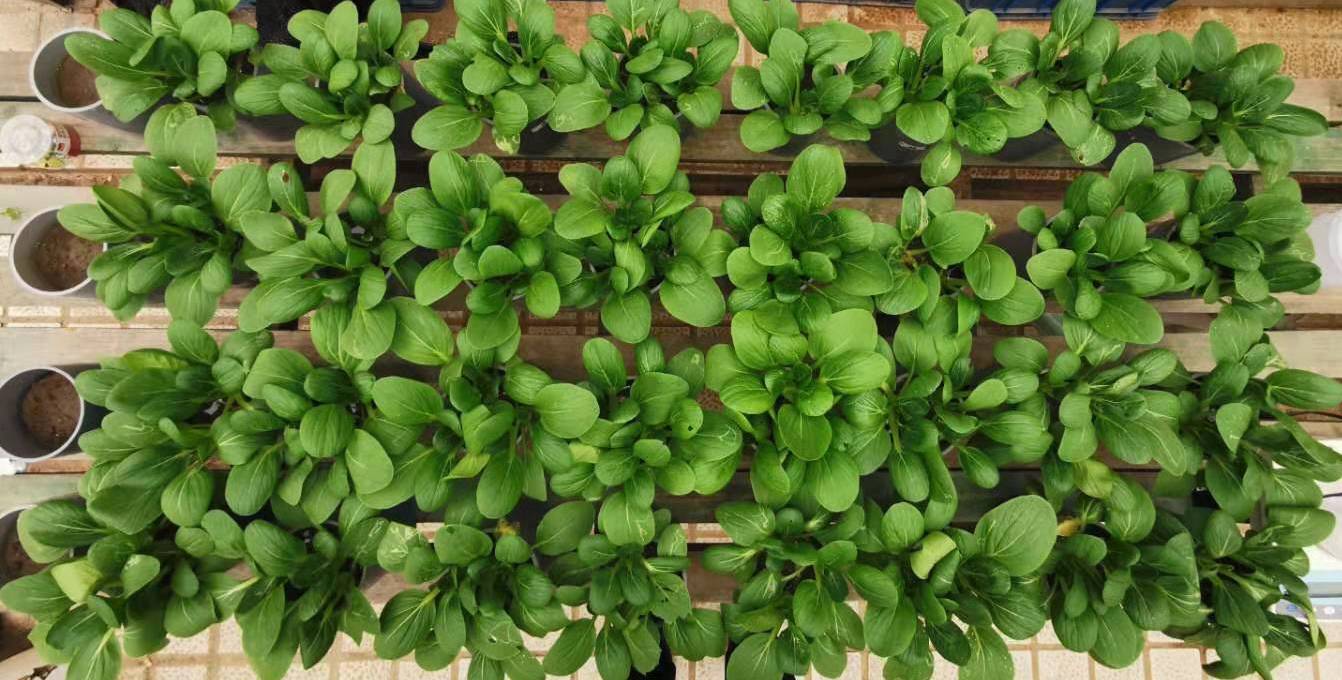


**(a)**

**(b)**





FIGURE S6. The effect of fulvic acids on the fresh yield, water content and shoot dry biomass of pakchoi plants cv. *Gailiangjinpin28* grown in soil cultures for 20 (a, c and e) and 33 days (b, d and f). Data are means±s.e (*N=4*). The same letters denote insignificant differences (*P*<0.05) among treatments according to Duncan’s new multiple range test.
